# Higher chronic stress is associated with a decrease in temporal sensitivity but not in subjective duration in healthy young men

**DOI:** 10.3389/fpsyg.2015.01010

**Published:** 2015-07-21

**Authors:** Zhuxi Yao, Jianhui Wu, Bin Zhou, Kan Zhang, Liang Zhang

**Affiliations:** ^1^Key Laboratory of Behavioral Science, Institute of Psychology, Chinese Academy of Sciences, Beijing, China; ^2^University of Chinese Academy of Sciences, Beijing, China

**Keywords:** chronic stress, temporal sensitivity, temporal bisection, subjective duration, time perception

## Abstract

Maintaining accurate and precise temporal perception under conditions of stress is important. Studies in animal models and clinic patients have suggested that time perception can change under chronic stress. Little is known, however, about the relationship between chronic stress and time perception in healthy individuals. Here, a sample of 62 healthy young men completed Cohen’s Perceived Stress Scale (PSS) as a measure of chronic stress levels, while time perception was measured using a temporal bisection task. This task used short (400 ms) and long (1600 ms) visual signals as anchor durations. Participants were presented with a range of intermediate probe durations and were required to judge whether the durations were more similar to the short or the long anchor. Results showed that chronic stress was negatively related to temporal sensitivity indexed by the Weber ratio. However, there was no significant correlation between chronic stress and subjective duration indexed by the bisection point. These results demonstrate that higher chronic stress is associated with lower temporal sensitivity and thus provide evidence for a link between chronic stress and time perception in healthy adults.

## Introduction

Time perception is fundamental to behaviors that are essential for survival and adaptation, such as conditioning reflexes (Gallistel and Gibbon, 2000), speech recognition (Nourski and Brugge, 2011), and motor control (Buhusi and Meck, 2005). Maintaining temporal accuracy and precision is even more important under conditions of stress (Hancock and Weaver, 2005). However, time perception seems vulnerable to stress (Meck, 1983; Droit-Volet et al., 2010). Anecdotally, for individuals under chronic stress, time may feel either slow and “dragging” or fast and “in a rush.” The relationship between chronic stress and time perception, however, has scarcely been studied.

Subjective duration and temporal sensitivity are two key indicators of different aspects of time perception (Meck, 1983; Grondin and Rammsayer, 2003). Subjective duration refers to one’s perceived duration of a stimulus. Temporal sensitivity refers to one’s ability to discriminate different stimuli durations. The temporal-bisection task is one of the most commonly used time perception tasks, and it can measure both subjective duration and temporal sensitivity (Allan and Gibbon, 1991; Wearden, 1991; for reviews, see Kopec and Brody, 2010; Wearden and Jones, 2013). The index of subjective duration in a temporal-bisection task is the bisection point (BP), which indicates the interval perceived to be equidistant to two anchors, with lower BPs indicating longer subjective durations (Droit-Volet et al., 2010, 2011). The index of temporal sensitivity in a temporal-bisection task is the Weber ratio (WR), with higher WR values indicating lower temporal sensitivity in discriminating different durations (Kopec and Brody, 2010; Wearden and Jones, 2013).

Cognitive models of time perception assume that time perception is based on three processing stages: the clock stage, memory stage, and decision stage (Church, 1984; Zakay and Block, 1997; Grondin, 2010; Kopec and Brody, 2010). In the clock stage, attention and arousal are two important mechanisms for the initial encoding of time (Zakay and Block, 1997). In the memory stage, the representations of durations are stored in working memory and/or long-term memory, and so unimpaired working memory and long-term memory are necessary for accurate and precise time perception (Wearden et al., 1997; Pouthas and Perbal, 2004; Roy et al., 2012). In the decision stage, responses are made according to some decision rule (Wearden, 1991).

Under stressful conditions, individuals usually show increased arousal (McEwen, 2004). Ample evidence has suggested that stress affects the cognitive components supporting time perception, such as attention (Caswell et al., 2003; Liston et al., 2009), working memory (Mika et al., 2012; Corrêa et al., 2015), and long-term memory (Cho, 2001; Lovell et al., 2014; Corrêa et al., 2015). Furthermore, brain regions, including prefrontal cortex and hippocampus, which are involved in time perception (Lewis and Miall, 2003, 2006; Meck, 2005), are also sensitive to the effects of stress hormones (McEwen, 2004; Ulrich-Lai and Herman, 2009). For example, the dorsolateral prefrontal cortex is involved in the timing of long intervals (Lewis and Miall, 2006), and the functioning of this region is affected by acute stress (Qin et al., 2009). Thus, altered time perception under stress is probable.

Changes in time perception have been reported under acute stress. Previous studies found subjective durations to be longer under acute stress, which was interpreted as due to increased arousal (Meck, 1983; Watts and Sharrock, 1984; Droit-Volet et al., 2010; Tamm et al., 2014). Temporal sensitivity also changes under acute stress, with the particular direction associated with the nature of the acute stressors used in different studies (Watts and Sharrock, 1984; Droit-Volet et al., 2010; Tamm et al., 2014). For example, the temporal sensitivity was decreased under psychological stress (Watts and Sharrock, 1984), but increased or unchanged under physiological stress, such as an extremely hot environment (Tamm et al., 2014) or loud white-noise stress (Droit-Volet et al., 2010).

In contrast with acute stress, chronic stress is more frequently related to cognitive and behavioral problems as well as maladaptive changes in brain structures (Selye, 1956; McEwen, 2004). Acute stress and chronic stress are distinguished by the duration of the stressors, with acute stress lasting from minutes to hours and chronic stress usually lasting 30 days or more (Stoney et al., 1999). Cognitive processes supporting time perception, such as attention (Caswell et al., 2003; Liston et al., 2009), working memory (Mika et al., 2012; Corrêa et al., 2015), and long-term memory (Cho, 2001; Lovell et al., 2014; Corrêa et al., 2015), have been shown to be impaired in chronically stressed individuals. Furthermore, prefrontal neuronal structures and hippocampus, which underlie time perception (Lewis and Miall, 2003, 2006; Meck, 2005), can atrophy under chronic stress (for a review, see McEwen, 2004). The results from animal studies have shown decreased temporal sensitivities but unchanged subjective durations in rats that experienced chronic foot-shock stress (Meck, 1983). Clinical research has found that patients with disorders associated with chronic stress show prolonged subjective durations (Bschor et al., 2004; Wittmann et al., 2006) or less precise time estimation (Kowalski et al., 2012). Little is known about the relationship between chronic stress and time perception in healthy individuals.

The current study investigated the relationship between chronic stress and time perception in healthy adult men. Cohen’s Perceived Stress Scale (Cohen and Williamson, 1988) was used to measure chronic stress levels, and time perception was assessed using a temporal-bisection task (Droit-Volet et al., 2010, 2011). We hypothesized that chronic stress would be associated with changes in time perception, which would be demonstrated by decreased temporal sensitivity and/or altered subjective durations.

## Materials and Methods

### Participants

Sixty-two male students, aged 18–24 years (mean = 21.24, SD = 1.31), were recruited from universities in Beijing by advertising online. Only male students were included to prevent the confounding influence of sex on stress effects (Luine, 2002; Backovic et al., 2012). Due to their potential influence on stress responses, the following exclusion criteria were also employed: (1) the chronic use of any psychiatric, neurological, or endocrine medicine; (2) any major chronic physiological disease; (3) any history of psychiatric or neurological disorders; and (4) chronic overnight work or irregular circadian rhythm. All participants were right handed by self-report. All participants gave written informed consent and were paid for their participation. This experiment was approved by the Ethics Committee of Human Experimentation in the Institute of Psychology, Chinese Academy of Sciences.

### Chronic Stress Measure

Chronic stress was assessed with the Chinese version of the 10-item Perceived Stress Scale (PSS-10; Cohen and Williamson, 1988; Wang et al., 2011), which has achieved excellent internal consistency and test–retest reliability (Wang et al., 2011). For the internal consistency reliability in the present data set, Cronbach’s alpha of the PSS-10 was 0.62 for the whole scale. The PSS measures how often participants felt that life was overwhelming, uncontrollable, and unpredictable over the last month (Cohen et al., 1983), and it has been widely used to measure chronic stress in clinical and research settings (Gianaros et al., 2007; Liston et al., 2009; Wang et al., 2011; Boals and Banks, 2012; Lovell et al., 2014). Scale responses range from 0 (*never*) to 4 (*very often*), with higher scores indicating higher levels of chronic stress.

### Stimuli and the Temporal-bisection Task

The settings and procedure of the temporal-bisection task were similar to that of previous studies (Droit-Volet et al., 2010, 2011). The stimulus for timing was always a blue circle (2.5 cm in diameter) presented in the center of the computer screen with a viewing angle of approximately 2° (participants’ viewing distance from the monitor was approximately 70 cm). The short (S) and long (L) anchor durations were 400 and 1600 ms, respectively. The probe durations were 400, 600, 800, 1000, 1200, 1400, and 1600 ms.

In the temporal bisection task, participants were first presented with S and L anchors once in a random order. Then, they completed eight blocks of seven trials (one trial for each probe duration). The probe durations were presented randomly within each block. For each trial, the word “ready” was first presented for 500 ms, immediately followed by a 200-ms interval and then the stimulus (the blue circle) for timing. Participants were required to judge whether the probe duration was more similar to the S anchor or the L anchor by pressing the corresponding key with the index fingers of their right or left hand. The inter-trial interval was randomized between 500 and 1500 ms in duration. All participants were instructed not to count time, as the counting of time would affect temporal performance (Clement and Droit-Volet, 2006).

### Data Analyses

For the behavioral performance of time perception, we first computed P(long), the proportion of “long” responses for each probe duration. For example, if 25% of the 600-ms probes were judged to be long, the P(long) for the 600-ms probe would be 25%. The logistic function, *P*(*long*) = 1/[1 + *exp*(*a* × *Duration* + *b*)], was then fitted to individual-participant data, in which *Duration* stood for probe duration, and *a* and *b* were free parameters. Two indices of time perception, the BP and the WR, were derived from the parameters of this logistic function (Droit-Volet et al., 2010). BP was defined as the probe duration that gave rise to 50% “long” responses, i.e., *BP* = *Duration*(*P*(*long*) = 50%) = –*a*/*b* (Meck, 1983). The BP indicates the subjective duration, i.e., a lower BP suggests a longer subjective duration. In order to calculate the WR, the probe durations that gave rise to 25 and 75% “long” responses, [*Duration*(*P*(*long*) = 25%)] and [*Duration*(*P*(*long*) = 75%)], were first obtained. The WR was calculated by the following expression, *WR* = [*Duration*(*P*(*long*) = 75%) – *Duration*(*P*(*long*) = 25%)]/(2 × *BP*) = {[ln(1/3) – *b*]/*a* – [ln(3) – *b*]/*a*}/(2 × –*a*/*b*) (Meck, 1983). The WR indicates temporal sensitivity, i.e., a lower WR value indicates a higher temporal sensitivity.

For the relationship between chronic stress and time perception, Pearson correlations were calculated between PSS scores and temporal behaviors, including BP and WR. Reported *p*-values are two-tailed.

## Results

**FIGURE 1:**
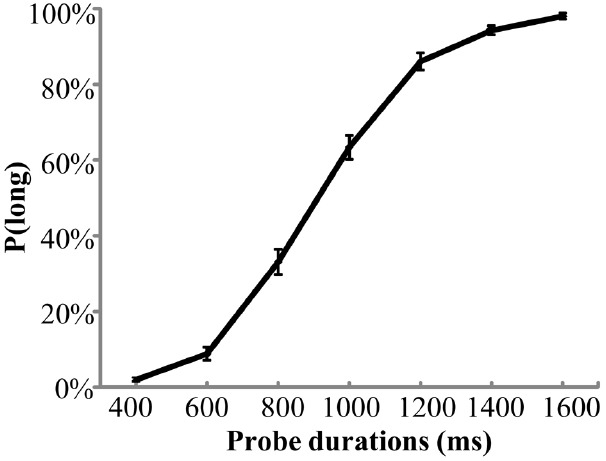
**Mean proportion of “long” responses, P(long), plotted against probe durations for the temporal bisection task.** Error bars indicate SEM.

The mean value of PSS scores for all participants was 15.5 ( ± 3.4), which was similar to previous studies in healthy young populations (Cohen and Williamson, 1988). Figure 1 shows averaged P(long) across all participants plotted against probe durations. A one-way ANOVA of P(long) showed a significant main effect of duration indicating that P(long) increased as probe duration increased, *F*(6,360) = 481.8, *p* < 0.001, partial η^2^ = 0.888. For subjective durations, the mean value of BP was 941 ( ± 150) ms. For temporal sensitivity, the mean value of WR was 0.14 ( ± 0.06).

**FIGURE 2:**
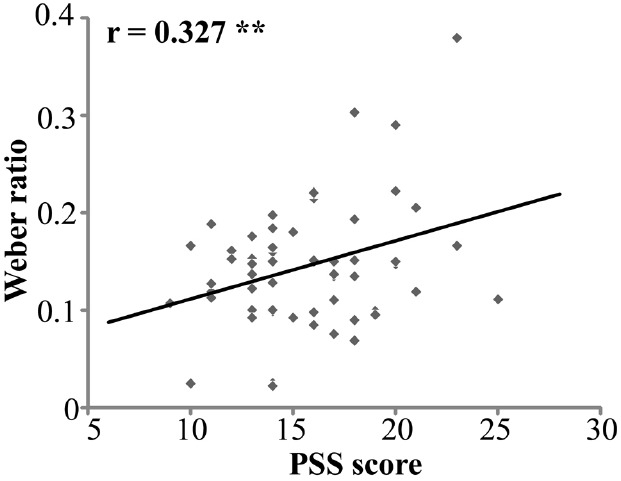
**Correlation of PSS scores and Weber ratio (*n* = 62).** PSS stands for Perceived Stress Scale. **p < 0.01.

For the relationship between chronic stress and time perception, the Pearson correlation between PSS scores and BP was not significant (*r* = –0.115, *p* = 0.374). However, the positive correlation between PSS and WR was significant, *r* = 0.327, *n* = 62, *p* < 0.01 (Figure 2).

## Discussion

The current study investigated the relationship between chronic stress and time perception in healthy adult males. PSS scores were positively correlated with the WR, indicating that higher levels of chronic stress were associated with reduced temporal sensitivity. However, PSS scores were not correlated with subjective duration indexed by the BP.

The positive correlation between PSS scores and WR suggested that higher chronic-stress levels were associated with lower temporal sensitivity. This finding is consistent with previous animal studies of chronic stress that found decreased temporal sensitivity in rats exposed to chronic foot-shock stress (Meck, 1983). Furthermore, clinical studies have found that patients with posttraumatic disorder exhibited less precise time estimation compared to controls (Kowalski et al., 2012). The present study extends these previous findings by showing a negative association between temporal sensitivity and chronic stress in healthy adults.

Ample evidence suggests that chronic stress is associated with deficits in attention (Caswell et al., 2003; Liston et al., 2009; Yuan et al., 2014), working memory (Mika et al., 2012; Corrêa et al., 2015), and long-term memory (Lovell et al., 2014; Corrêa et al., 2015), as well as atrophy and dysfunctional changes in corresponding brain regions, such as the prefrontal cortex and hippocampus (McEwen, 2004; Ulrich-Lai and Herman, 2009). These neural functions are critical to the cognitive mechanisms underlying temporal sensitivity (Church, 1984; Wearden et al., 1997; Zakay and Block, 1997; Grondin, 2010; Zelanti and Droit-Volet, 2011; Droit-Volet and Zelanti, 2013), and their impairment under chronic stress may explain the negative correlation between chronic stress and temporal sensitivity.

In addition, neurochemical studies have found suppressed dopaminergic transmission in the brain under chronic stress (Gambarana et al., 1999; Mizoguchi et al., 2000; Lammel et al., 2014). While dopamine-mediated mechanisms have been consistently implicated in temporal perception (Meck, 1983, 2006; Coull et al., 2011; Narayanan et al., 2012), studies have found that dopaminergic antagonists reduced temporal sensitivity (for a review see, Coull et al., 2011). Thus, reduction in dopaminergic transmission under chronic stress might contribute to the poorer temporal sensitivity of the individuals who were experiencing higher chronic stress.

Subjective duration as indexed by BP values did not correlate with PSS scores. This result is also consistent with studies of animal models (Meck, 1983), suggesting that subjective durations can remain unchanged despite stress-related decreases in temporal sensitivity. The judgments of probe durations in temporal-bisection tasks are made by comparing probe durations with one’s representations of anchor durations (Wearden and Jones, 2013). It might be the case that participants with different chronic-stress levels have different internal clock speeds, but that these differences similarly influence representations of both the anchor and probe durations.

There are a number of limitations to our study. First, since we did not and cannot manipulate chronic stress in participants, it is impossible to make causal inferences in the current study. Intuitively, however, it seems more likely that chronic stress impaired temporal sensitivity, probably via neural mechanisms similar to those underlying the association between chronic stress and impaired cognition. Second, only male adults participated in the present study. Whether the current finding can be generalized to female populations is still unclear. Third, the range of durations in the current temporal-bisection task was 400 to 1600 ms, and thus the results of the present study may not generalize to the perception of all time durations. Indeed, different mechanisms have been proposed for the processing of temporal information of different durations (Lewis and Miall, 2006). Fourth, no index of attentional ability, working memory, or long-term memory was included in the present study. It has not yet been determined which mechanism or mechanisms play a role in the association between chronic stress and time perception.

In summary, the present study found that higher chronic stress was associated with lower temporal sensitivity, while there was no such result for subjective duration. This replicates previous findings in animal models and provides evidence for the link between chronic stress and time perception in healthy human populations.

## Author Contributions

JW, KZ, and LZ conceived and designed the experiment; ZY performed the experiment and collected data; ZY, JW, and LZ analyzed data; BZ contributed to the analysis and interpretation of data on time perception; ZY drafted the manuscript; JW, BZ, KZ, and LZ provided critical revisions. ZY, JW, BZ, KZ, and LZ approved the final version of work and agreed to be accountable for all aspects of the work.

### Conflict of Interest Statement

The authors declare that the research was conducted in the absence of any commercial or financial relationships that could be construed as a potential conflict of interest.

## Abbreviations

BP: the bisection point, which is the index of subjective duration in a temporal-bisection task
PSS: Cohen’s Perceived Stress Scale
WR: the Weber ratio, which is the index of temporal sensitivity in a temporal-bisection task.
